# Feedback on video recorded consultations in medical teaching: why students loathe and love it – a focus-group based qualitative study

**DOI:** 10.1186/1472-6920-5-28

**Published:** 2005-07-19

**Authors:** Stein Nilsen, Anders Baerheim

**Affiliations:** 1 Section for General Practice, Department of Public Health and Primary Health Care, University of Bergen, Kalfarveien 31, N-5009 Bergen, Norway

## Abstract

**Background:**

Feedback on videotaped consultations is a useful way to enhance consultation skills among medical students. The method is becoming increasingly common, but is still not widely implemented in medical education. One obstacle might be that many students seem to consider this educational approach a stressful experience and are reluctant to participate. In order to improve the process and make it more acceptable to the participants, we wanted to identify possible problems experienced by students when making and receiving feedback on their video taped consultations.

**Methods:**

Nineteen of 75 students at the University of Bergen, Norway, participating in a consultation course in their final term of medical school underwent focus group interviews immediately following a video-based feedback session. The material was audio-taped, transcribed, and analysed by phenomenological qualitative analysis.

**Results:**

The study uncovered that some students experienced emotional distress before the start of the course. They were apprehensive and lacking in confidence, expressing fear about exposing lack of skills and competence in front of each other. The video evaluation session and feedback process were evaluated positively however, and they found that their worries had been exaggerated. The video evaluation process also seemed to help strengthen the students' self esteem and self-confidence, and they welcomed this.

**Conclusion:**

Our study provides insight regarding the vulnerability of students receiving feedback from videotaped consultations and their need for reassurance and support in the process, and demonstrates the importance of carefully considering the design and execution of such educational programs.

## Background

During the last couple of decades, the importance of good patient-doctor communication has been increasingly emphasized, and teaching communication skills in medical school and post graduate courses is no longer a novelty. There is increasing evidence to suggest that this educational input results in an overall improvement in the communication skills of medical students and doctors, especially where the training includes some form of feedback on the trainees' performance [1,2].

One educational approach involves videotaping of consultations between medical student/ doctors and simulated or real patients, and providing personal feedback from others through later assessment of the taped consultations. Feedback on videotape or after direct observation has been shown to enhance development of both general communication skills and more specific consultation techniques [3,4]. This kind of feedback has also been demonstrated to have a more lasting impact on the students' communications skills than conventional education such as lectures or textbooks only, and it has been recommended that all medical students should be provided feedback training [5].

Despite this evidence only a minority of communication training programs provide this kind of individual feedback [6]. A common experience among medical teachers is that students or doctors participating in video-based training programs seem to be very apprehensive beforehand and consider this educational approach to be stressful [7]. This may represent an obstacle to implementation. The few studies assessing this mode of communication teaching have made use mainly of questionnaires or other quantitative research methods to collect information [6,7].

Roter et al did a pre/post comparison of residents' performance after undergoing an innovative video feedback programme. They used a coded, quantitative analysis to demonstrate this method as a powerful and effective teaching tool [6].

In another study, Paul at al made an assessment of the feasibility of video feedback in teaching clinical skills, and of the students' own perception of the effectiveness of this training. They used observer assessment and semi-structured interviews, making a quantitative rating of the students' performance. Among their findings was that feedback on video performances can be useful and effective for improving clinical skills. They also added some open questions, and in this part the students' apprehension before the course became evident [7].

We judged focus group interviews to be the most suitable assessment tool for our purpose, as we believed it would provide more detailed and rich insights into these questions. We wanted to explore this issue in more detail, in order to deal with it more effectively and make the process more acceptable to the participants.

This study explores students' experiences of receiving feedback on their videotaped consultations, and aims to provide insight about how this element of their education can be refined and further developed.

## Methods

Communication and consultation training in Bergen University Medical School is partly given as courses in year 3 and 5, but the main emphasis takes places during the final term of the 6 year curriculum, where the main focus is on general practice. The students have a 4-week training period in a GP practice, and also take a course that focuses on different aspects of the consultation process. The course consists of two parts, the first containing practical exercises, group discussions, demonstrations and lectures. In the second part, which has been evaluated in this study, each student videotapes a consultation with a real patient. This is the first time the students' consultations have been videotaped. The consultation takes place at Bergen's community- based Emergency Department, where acutely ill patients from the inner city attend for advice and treatment. Informed consent to videotape the consultation and show it in a closed group of students is obtained from the patients prior to the consultation. The student can, if needed, seek advice and support from a teaching doctor during the consultation.

A few days later the students meet in groups of 6 or 7, led by a mentor experienced in general practice, and also trained in this type of group leadership. The videotapes are reviewed and analysed one at a time, inspired by the ALOBA (Agenda-led, outcome-based) guidelines [8], as follows: Before showing his/her video, the student is encouraged to define critical incidents and/or points of special interest. He/she is asked to appoint a "critical friend", who is assigned to give specific feedback on areas for possible improvement. The other students are also given specific roles in the feedback process, each paying attention to a certain aspect of the consultation. The tape is played back, generally in its entirety, after which the student shares his own assessment of his performance with the group. He/she is invited to define an agenda for discussion, in which the whole group then takes part, seeking to provide balanced feedback, focusing both on what works and areas where improvements could be made. There is opportunity for role-play, focusing on particular points of interest in the consultation, and critical incidents from the tape may be reviewed. The students themselves are encouraged to give one another feedback while the mentor supervises the process, providing comments or further advice when needed. The time allowed for the review session of each student is approx 45–60 minutes, giving a total of 6 hours. The tapes are erased immediately following the feedback session.

In our study, three video review groups out of a total of 12 (19 students out of 75), were invited to participate, and all consented. Their average age was 27.1 years, 58% were males, and all were of Norwegian descent. The members of the groups did not differ significantly from the general student cohort. The students were informed about the purpose of the interview, and given the opportunity to withdraw if they wished. They were also encouraged to observe confidentiality about the group discussion. Written consent to participate was not obtained. The interviews took place immediately after finishing the video feedback sessions.

The interviews were conducted as focus group interviews, as described by Kvale [9]. This approach was chosen for its suitability for collecting qualitative data from a group, especially when assessing attitudes, experience and values in an environment where individuals act or work together. There were 6 or 7 members in each, a size considered ideal for a focus group interview. The interviews were audio-recorded, and the interviewer was supported by an assistant, who took notes. Each interview lasted for 60–90 minutes. The interviews were afterwards transcribed fully by the interviewer. The text was then analysed in a qualitative mode, as described by Giorgi, modified by Malterud [10].

First, the transcripts were read, to get a general overview of the topics commented upon. They were scrutinized to identify all text elements concerning the different aspects of the consultation and the feedback. Each element was coded according to topic or type of factor. The codes were derived from the data, not decided beforehand. Similarly coded elements were interpreted for a common meaning, and were then summarized using expressions close to the students' own words.

The interviewer (SN), a general practitioner, analyzed the focus group interview text and drafted the manuscript. AB made a separate analysis of large parts of the text, and agreement was reached through discussion where differences in analysis appeared.

## Results

From the text analysis 3 major themes appeared: Concerns, the feedback process and reassurance.

### Concerns

A major theme in the students' evaluation dealt with their apprehension and anxiety prior to the video taped consultations and feedback group discussion. Their concerns evolved around both procedural elements and the feedback process itself.

Among their concerns were:

• carrying out a consultation in an unfamiliar setting or while being videotaped

• embarrassment at watching themselves on videotape together with fellow students

• fear of being shown up as lacking in medical knowledge

• fear of being thought to be inadequate in personality, or in basic communication skills

• fear that if they were judged incompetent at this late stage of their medical training, there would be inadequate time for improvement

One student, seemingly confident and experienced, expressed his worries this way: *"The worst thing that could happen would be to demonstrate a complete lack of medical knowledge, to miss something obvious and crucial. "...That, I feel, would be a real blow to me " (male, 25 y, 2. interview)*. Another student commented that medical students in general worry more than other students about being unsuccessful: *"... This is their nature, and to imagine making a video of your own failures is frightening"(female, 27 y, 3.interview)*. Other concerns were also expressed: One student voiced doubts as to whether specific consultation skills he felt obliged to demonstrate would not be appropriate for the consultation being videoed, and feared he would be criticized for not using these techniques. Another mentioned that being first to show her video made it more embarrassing, it would have been easier to have appeared later in the session.

### The feedback process

Some of the respondents stated that despite feeling apprehensive before the start of the course, they experienced few problems once they got into it; and that eventually they realized that most of their fears had been groundless. One student who had technical problems playing back her tape put it this way: *"I was not happy with my consultation and hoped that I might somehow lose it. When this actually happened I came to regret it, because I realized afterwards that getting feedback from the others would in fact have been a useful experience for me."(female, 25 y, 2. interview)*

Some students gave positive comments on the feedback, as follows:

• the advice I got about what could be improved was being worded carefully and with respect, and did not make me think less of myself afterwards

• feedback always ended with a positive conclusion

• feedback was expressed in a constructive manner, so that possible improvements could be pointed out without anybody losing face.

One student mentioned that she found it easy to agree with the advice given regarding possible improvements, because she had already been able to observe her own areas for development while watching the tape. Another mentioned that although he didn't agree with all the feedback he was given, he still found the discussion valuable. Others described the group environment as feeling safe and protective. They gave several different reasons for this:

• few students in each group

• everybody sharing the same experience

• all showing a positive attitude and willingness to learn from each other

• this type of session provides an environment which is conducive for accepting criticism

Some students found the rules guiding the feedback helpful. One mentioned the way each of them was given a specific role for every new round of feedback, and emphasized the point that the student receiving feedback himself appointed his main "opponent". Another student approved of the expressed aim to make the feedback specific and detailed in order to be of best possible use to the recipient. Some students particularly liked the fact that most of the feedback was given by the students themselves, while the group leader had more of a supervisory role, supplying additional comments where appropriate.

### Reassurance

Before watching the videotapes, some of the students said that they did not think their own consultations had gone very well. They admitted to being very self-critical and, due to their limited professional experience as doctors, insecure and vulnerable. Had the feedback from the group been insensitive or harshly-worded, the effect could have been damaging.

After having watched the tapes they were relieved to find that they in fact had done better than expected, and were reassured to have this confirmed by their fellow students.

A number of points were made by the students, following the session. For some, reassurance regarding their professional ability, and more specifically their consultation skills, was of paramount importance. Another put it this way: *"Most of the students got positive feedback, as I did myself. I was happy about that: I don't find it easy to take a negative response to something I have put so much of myself into." (Male, 28, 1.interview)*.

The importance of treating each student individually, so that each is allowed to develop his own personal style was also mentioned. One noted that the session highlighted the fact that many different approaches can be valid, and he learnt a lot by observing others.

One student felt that carrying out one single consultation only added to the stress, since this left no opportunity to become familiar with the situation, and demonstrate improvement the next time. It was suggested that for the future, a number of consultations should be taped.

## Discussion

This study showed that consultation training by way of video taping real consultations with subsequent feedback was in general considered acceptable, useful and inspiring by the participating students. However, they experienced a considerable amount of anxiety and apprehension before and during the course, resulting in a strong need for reassurance and a positive evaluation. The feedback process seemed to meet this need, and was described as respectful, easing unwarranted fear, increasing self -esteem and contributing to personal growth of these emerging doctors.

This study highlights aspects that have been noted, but not widely discussed in many earlier studies. An important aspect is the level of anxiety and feeling of vulnerability suffered by the students in this particular educational setting. This initial apprehension by trainees has been noted by others: Paul et al found that the majority of their students reported anxiety and resistance to videotaping, but that their inhibition diminished with increasing practice and experience [7]. Most students in their study believed that they would have gained confidence had they had the opportunity to view a few videotapes of standard consultations before making their own. Smith et al noted the same initial wariness in neurology trainees undergoing a similar educational process, and concluded that doctors may not yet be ready to accept these methods of training without first becoming more familiar with them [12]. However, Rees et al found that undergraduate students preferred experiential methods of learning communication skills to more conventional methods such as lectures, and their students did not emphasize the stressfulness of the experience [13]. One reason for this may be that their students are familiar with making video consultations form their first year of medical school (C. Rees, personal communication, 2005). An important practice implication of this study is that students should be introduced to such teaching methods from the start of their training rather than waiting until later stages, when the stakes are so much higher. Practice in safe environments such as clinical skills resource centres and with simulated patients would also possibly lessen the burden.

In our study we found that some of the observed anxiety and stress among the students was due to the fact that the recorded consultations were made in an environment completely unknown to them, and we suppose that recording the video in a more familiar setting would lessen this burden. This would be achievable if the consultations were taped during primary care training, or if the students were given the opportunity of a second performance after the initial feedback [14].

Many of the students admitted afterwards that their initial worries had been groundless, indicating that they had a misguided impression of the process beforehand. It should be possible to decrease their apprehension by paying more attention to the general information provided, and to specific preparation prior to the course. Another way of dealing with the fear of hostile criticism from others, is to allow the students to choose their own groups. This, however, could risk a too "friendly" environment where important criticisms are less likely to surface. Mavis et al suggest instead that students should be grouped randomly, instead of choosing their own group members, to counteract the observation that students were reluctant to provide less favourable feedback to peers [15].

Many students showed a very self-critical attitude towards their own performance and expressed genuine satisfaction and encouragement from receiving positive evaluation of their consultations. This desire for recognition and reassurance was also noted by Lings and Gray in a study of British GPs participating in an educational program to promote higher standards of care [16]. The doctors in this program were considered likely to be among the most confident to begin with, but still had a strong need for reassurance [17]. One would assume that this kind of support is even more important for students at the start of their careers. Well-earned personal encouragement may provide valuable inspiration to further development of communication skills, which may otherwise often be given low priority among all the other challenges a young doctor is about to face. The students' fears of appearing incompetent must be treated with respect, and mentoring of the groups and the adhesion to the guidelines ruling the feedback process should be given careful consideration. The mentor's role and responsibility in this educational process has not been given much attention so far, and should be investigated further.

The way in which the feedback sessions were conducted has been criticized in prior evaluations of this education model [6]. Among problems observed are superficial reviews of the tapes and difficulties in identifying critical incidences. The problems seem related to the time and cost of faculty training and preparations. To meet this challenge, different approaches have been developed. The method chosen for reviews of the tapes in our program, based on the ALOBA guidelines described earlier [8], seemed to work well, and was praised by many students. One of the keys to success might be the rather detailed guiding rules which the process adhered to, giving each student a specific role and a guide for delivering feedback. This provided a feeling of safety within the group and also gained attention for each part of the consultation, securing a balance between positive and corrective judgment. However, a possible drawback may be that students tend to overestimate the performance of their fellow students [15]. This puts a further responsibility on the mentor to ensure that the feedback is well-balanced.

The students groups interviewed were selected in such a way that each of the three groups had a different mentor, minimizing the possible effect of a specific mentor's personal impact. 19 of the 75 participating students (25%) were interviewed, securing a sufficiently broad sample of opinions. All students invited to participate did so: there were no refusals or withdrawals. The students knew each other well and appeared secure and relaxed in the interview setting, indicating that the interview group provided an environment where they could speak out freely. They were also granted anonymity as to the content of their given opinions. These factors may lessen an intra-group trend towards conformity which could otherwise present a problem, since participants who are unsure or alien to the group may tend to voice agreement with opinions already expressed by others [11]. The interviews were conducted immediately after the video feedback sessions, leaving no time for possible reflection and second thoughts about the experience. After a long and intense session, the students may have been tired and anxious to get away, and less likely to put much thought into their answers. On the other hand, carrying out the interviews straight away contributed to full participation from the students. The experience was fresh in their minds, perhaps resulting in a response more immediate than it would have been at a later stage, and therefore enhancing the validity of their comments.

The students were asked to participate while they were in a group doing the video feedback review, and the focus interview was carried out right afterwards. They did not get a written invitation prior to this, and therefore had limited opportunity to consider the invitation and refuse to participate. This might represent a coercion to participate, and could help explain a 100% participation.

The evaluation appeared to be overall very positive, and one might suspect that intrinsic factors would inadvertently facilitate this. One such factor might be that the interviewer (SN) also was a member of the faculty staff, and this could represent a potential coercion towards too positive views. SN had, however, only been a part time (20%) employee for 6 months when the interviews took place, and the majority of the students had never met him before the interview. Thus, the students had no reason to fear consequences of expressing a negative view. Also, an interview guide was used to secure varied approaches to the issues in question, and to encourage differing views. Another possible issue is the fact that the groups invited to be interviewed were not chosen in a truly randomly fashion, but for pragmatic reasons. Still, the selection was made in a way that should make them representative of the whole cohort.

The students' general enthusiasm found here is also in agreement with the results of the written free text assessment that all students attending this course carry out after each teaching term. These written assessments have earlier been analysed formally [18].

## Conclusion

This study shows that students participating first time in peer feedback as part of their training in consultations, experience a high degree of anxiety and apprehension before and during the course; also demonstrating a strong need for reassurance and positive evaluation, both of their efforts and of their professional ability. The feedback process described here would appear to address this issue, and might contribute to the empowerment of the participants by increasing their confidence. This approach requires careful attention to design and procedure, in order to provide sufficient support for the students or trainees, both during the video recording sessions and the feedback process. The study also implicates that this educational approach should be introduced early in the medical school curriculum, in order to lessen the stress later.

## Competing interests

The author(s) declare that they have no competing interests.

## Authors' contributions

SN conducted the interviews, transcribed the text, performed the analysis and drafted the paper. AB was involved in the conception and design of the study, participated in analysis and edited the paper.

## Pre-publication history

The pre-publication history for this paper can be accessed here:



## References

[B1] Yedidia MJ, Gillespie CC, Kachur E (2003). Effect of communications training on medical student performance. JAMA.

[B2] Aspegren K (1999). BEME Guide no 2: Teaching and learning communication skills in medicine: a review with quality grading articles.

[B3] Holmwood C (1998). Direct observation: A primer for supervisors of doctors in training. Aus Fam Physician.

[B4] Beckman HB, Frankel RM (1994). The use of videotape in internal medicine training. J Gen Intern Med.

[B5] Maguire P, Fairbairn S, Fletcher C (1986). Consultation skills of young doctors: Benefits of feedback training in interviewing as students persists. Br Med J (Clin Res Ed).

[B6] Roter DL, Larson S, Shinitzky H (2004). Use of an innovate video feedback technique to enhance communication skills training. Med Educ.

[B7] Paul S, Dawson KP, Lanphear JH, Cheema MY (1998). Video recording feedback: a feasible and effective approach to teaching history-taking and physical examination skills in undergraduate paediatric medicine. Med Educ.

[B8] Kurtz SM, Silvermann JD, Draper J (1998). Teaching and Learning Communication Skills in Medicine.

[B9] Kvale S (1996). Interviews: An Introduction to Qualitative research Interviewing.

[B10] Malterud K (1996). Kvalitative metoder i medisinsk forskning.

[B11] Espeland A, Baerheim A (2003). Factors affecting general practitioners' decisions about plain radiography for back pain- a qualitative study. BMC Health Serv Res.

[B12] Smith PEM, Fuller GN, Kinnersley P, Brigley S, Elwyn G (2002). Using simulated consultations to develop communications skills for neurology trainees. Eur J Neurol.

[B13] Rees C, Sheard C, McPherson A (2004). Medical students' views and experiences of methods of teaching and learning communication skills. Patient Educ Couns.

[B14] Eaooskoon W, Sumawong V, Silpakit C (1996). Evaluation of training medical students in patient-interviewing skills by three modes of learning. J Med Assoc Thai.

[B15] Mavis BE, Ogle KS, Lovell KL, Madden LM (2002). Medical students as standardized patients to assess interviewing skills for pain evaluation. Med Educ.

[B16] Lings P, Gray DP (2002). Professional development for general practitioners through Fellowship by Assessment. Med Educ.

[B17] van Dalen J (2002). Assessment practices undermine self-confidence. Med Educ.

[B18] Barheim A, Meland E, Schei E (2000). Evaluating the patient-centered consultation course in Bergen. Tidsskr Nor Legeforen.

